# Effects of a Korean Traditional Dance Program on Health-related Fitness and Blood Lipid Profiles in Korean Elderly Females

**Published:** 2018-01

**Authors:** Su-Mi KIM, Hyun-Jeong PARK, Byung-Ju MIN, Wi-Young SO

**Affiliations:** 1. Dept. of Physical Education, Sookmyung Women’s University, Seoul, Korea; 2. Dept. of Physical Therapy, College of Health and Welfare, Kyungwoon University, Gumi-si, Korea; 3. Sports and Health Care Major, Korea National University of Transportation, Chungju-si, Korea

## Dear Editor-in-Chief

As humans age, cardiovascular and respiratory functions decrease and there are negative effects on body composition, the musculoskeletal system, and the nervous system, resulting in various chronic diseases such as obesity, cardiovascular disease, stroke, and hypertension, along with decreases in the ability to manage the activities of daily life, physical fitness level, and exercise capacity (1, 2). Many studies recommend encouraging physical activity and regular exercise for the elderly, as this has been shown to elevate physical fitness level, improve chronic diseases, and prevent the loss of physical function (3, 4).

Since elderly people have particular physical and mental limitations, the type of exercise selected should be appropriate, allowing for joy and vitality in their remaining years. From this viewpoint, Korean traditional dance is an excellent form of exercise for the elderly as it consists of slow movements set to music. Unfortunately, no previous studies have investigated the effectiveness of this form of exercise. Therefore, this study aimed to examine the effects of a Korean traditional dance program on health-related fitness and blood lipid profiles in Korean elderly females.

The present study included 13 elderly females who visited the Y Senior Welfare Center in Seoul to undergo measurements of health-related fitness and blood lipid profiles in 2016. The participants were divided into the Korean traditional dance group (n=7; age, 78.0±1.5 yr; height, 157.1±2.3 cm; weight, 59.1±5.4 kg) and control group (n=6; age, 77.7±1.6 yr; height, 156.7±1.0 cm; weight, 58.8±4.6 kg) to compare the effects of the intervention program.

All participants provided written informed consent to participate in the study. The study was approved by Ethics Committee of the university. The participants underwent fitness tests and blood collection before and after the intervention. The exercise group participated in a Korean traditional dance program for 12 wk, whereas the control group was asked not to perform any special or regular physical activity outside of the activities of daily life for 12 wk.

The exercise group performed a Korean traditional dance for one hour three times per week for 12 wk. The program was modified for the elderly and consisted of a warm-up (10 min), main exercise (40 min), and a cool-down (10 min). The exercise intensity was gradually increased over time by setting the intensity at 40%–45% of the heart reserve rate (HRR) for the first four weeks, 45%–55% for wk 5–8, and 55%–60% for wk 9–12. The Korean traditional dance program consisted of the following steps: (a) Bal didim dong jak, (b) Chung a yeok gi and Chung a pul gi, (c) Duk suk mol gi and Duk suk pul gi, (d) Gin gangang sulrae, (e) Gosalee gguk gi, (f) Jateun gangang sulrae, (g) Jung gangang sulrae, (h) Jwijin saeggi nolee, (i) Moon yeol gi, (j) Nam seng a nolala, (k) Pal yeo migi, modified on the exercise protocol (5).

All data are presented as mean±standard deviation. The Kolmogorov-Smirnov test was performed to identify the normal distribution of the population. Independent t-tests were used to analyze differences between the dependent variables before and after the Korean traditional dance program. All analyses were performed by using SPSS (ver. 18.0, Chicago, IL, USA). Statistical significance was set at *P*<0.05.

Regarding the grip strength (*P*=0.037), back scratch (*P*=0.001), and 30-sec chair stand (*P*<0.001), the exercise group showed a statistically significant improvement compared to the control group (Table 1). In addition, the exercise group showed a statistically significant improvement in HDL-C levels compared to the control group (*P*<0.001) (Table 2).

**Table 1:** Changes in health-related fitness after 12 wk of the Korean traditional dance program

***Variables***	***Period***	***Group***		***t***	***P***
		***Exercise***	***Control***		
Grip strength (kg)	Pre	16.76±3.89	19.37±3.70	−1.236	0.242
	Post	20.54±2.92	16.55±3.16	2.366	0.037^*^
Sit-and-reach (cm)	Pre	28.93±7.25	24.50±5.13	1.248	0.238
	Post	33.14±8.67	24.83±4.75	2.086	0.061
Back scratch (cm)	Pre	5.00±2.38	3.00±1.55	1.758	0.107
	Post	10.00±4.40	1.83±0.98	4.429	0.001^**^
One leg balance with eyes close (sec)	Pre	3.71±1.80	5.83±2.93	−1.601	0.138
	Post	5.00±3.46	4.83±2.86	0.094	0.927
2-minute steps (reps)	Pre	82.86±9.79	91.33±7.89	−1.697	0.118
	Post	89.29±11.46	76.83±36.00	0.871	0.402
Chair stand (reps/30 sec)	Pre	12.86±1.95	13.00±1.90	−0.133	0.896
	Post	26.14±5.27	12.83±1.83	5.854	<0.001^***^

Data are presented as means±standard deviations

* *P*<0.05,

** *P*<0.01,

*** *P*<0.001; tested by Independent t-tests

**Table 2:** Changes in blood lipid profiles after 12 wk of the Korean traditional dance program

***Item***	***Period***	***Group***		***t***	***P***
		***Exercise***	***Control***		
TG (mg/d*ℓ*)	Pre	147.14±27.71	146.33±26.59	0.053	0.958
	Post	143.43±36.23	172.33±62.85	−1.037	0.322
HDL-C (mg/d*ℓ*)	Pre	80.86±7.10	73.17±10.15	1.63	0.137
	Post	83.29±5.77	58.33±4.59	8.521	<0.001^***^
TC (mg/d*ℓ*)	Pre	182.00±41.14	160.33±28.25	1.086	0.301
	Post	193.57±32.61	207.50±33.09	−0.763	0.462

Data are presented as means±standard deviations

TG; triglycerides, HDL-C; high density lipoprotein-cholesterol, TC; total cholesterol

*** *P*<0.001; tested by Independent t-tests

Twelve weeks of participation in a Korean traditional dance program might be effective to improve health-related fitness and blood lipid profiles in Korean elderly females.
